# Supplementary material for: Brownian motion data augmentation: a method to push neural network performance on nanopore sensors
Source: Bioinformatics. 2025 May 29;41(6):btaf323. doi: 10.1093/bioinformatics/btaf323 (PMC12208072; doi:10.1093/bioinformatics/btaf323)
Supplement: btaf323_Supplementary_Data [file btaf323_supplementary_data.zip › SI.pdf]

Brownian motion data augmentation: a method to push neural network performance on nanopore sensors (Supplementary Information)

Javier Kipen, Joakim Jalden

August 2024

1 Supporting information

1.1 Results of learning rate sweep in the neural networks trainings

All the results of the trainings for different learning rates (LR) and different configurations of the neural networks are shown in Table 1.

N Dense	Brow	Stretch eq	LR	Train acc	Test acc	N runs
[512,512]	No	No	5E-3	$95.91 \pm 1.83\%$	$88.98 \pm 1.83(93.97)\%$	54
[512,512]	No	No	2E-3	$99.27 \pm 0.01\%$	$92.86 \pm 0.16(94.91)\%$	108
[512,512]	No	No	1E-3	$99.35 \pm 0.01\%$	$92.96 \pm 0.12(95.11)\%$	208
[512,512]	No	No	5E-4	$99.16 \pm 0.02\%$	$92.76 \pm 0.14(94.79)\%$	150
[512,512]	No	No	3E-4	$98.80 \pm 0.03\%$	$92.82 \pm 0.13(94.94)\%$	148
[2048,1024]	No	No	2E-3	$99.32 \pm 0.02\%$	$92.45 \pm 0.16(95.07)\%$	159
[2048,1024]	No	No	1E-3	$99.22 \pm 0.02\%$	$92.85 \pm 0.13(95.14)\%$	212
[2048,1024]	No	No	5E-4	$99.15 \pm 0.02\%$	$93.04 \pm 0.11(95.24)\%$	260
[2048,1024]	No	No	2E-4	$99.07 \pm 0.03\%$	$93.00 \pm 0.17(95.30)\%$	114
[512,512]	Yes	No	1E-3	$98.18 \pm 0.01\%$	$93.12 \pm 0.12(95.22)\%$	235
[512,512]	Yes	No	5E-4	$98.00 \pm 0.02\%$	$93.22 \pm 0.30(95.34)\%$	51
[2048,1024]	Yes	No	1E-3	$98.67 \pm 0.02\%$	$92.94 \pm 0.20(94.75)\%$	68
[2048,1024]	Yes	No	5E-4	$98.68 \pm 0.01\%$	$93.43 \pm 0.10(95.69)\%$	349
[2048,1024]	No	Yes	5E-4	$99.01 \pm 0.01\%$	$93.04 \pm 0.09(95.27)\%$	395

Table 1: Learning rate sweep for different configurations of neural networks.

We tested several learning rates for the trainings without Brownian augmentation, both with higher and equal numbers of neurons in the last dense layers. The learning rates tested with Brownian augmentation were fewer since the aim was to demonstrate that a single configuration can achieve a higher accuracy. Finally, the stretch equivalent was only tested on the same configuration where Brownian augmentation demonstrated the highest test accuracy increase.

1.2 Confusion matrix of YupanaNet

Using the YupanaNet's model that achieved the highest barcode classification accuracy, we present in Figure 1 the corresponding confusion matrix.

In Figure 1, it can be seen that the average accuracy increased compared to QuipuNet. Particularly, the accuracy of the barcode "110" improved from 0.9 to 0.95. Although some barcodes, such as "100", experienced a slight decrease in accuracy, the overall classification performance of the model still improved.

1.3 Soft attention weights analysis

Interpreting the soft attention weights poses a challenge due to the large number of filters involved. In YupanaNet, the raw input signal is initially convolved with 64 distinct filters and subsequently processed through a batch normalization layer. The soft attention mechanism then generates a corresponding attention mask for each filter. Given the thousands of samples in our dataset, a comprehensive exploration of all results is unfeasible.

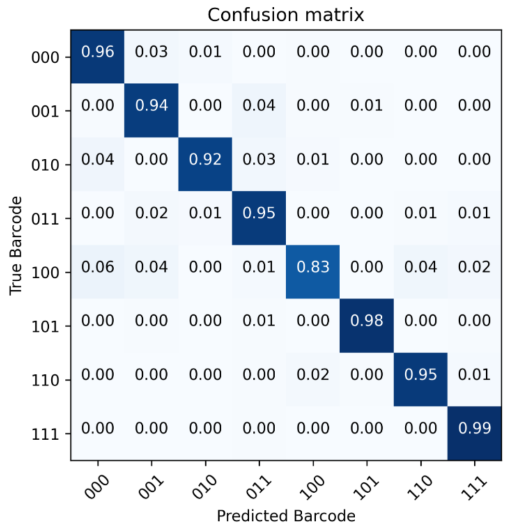

Figure 1: Confusion Matrix of Yupana Net

To gain insight into the behavior of the soft attention mechanism, we manually examined selected traces from the test set that were correctly classified by the best-performing YupanaNet model. Specifically, we extracted the outputs of the first convolutional layer (post-batch normalization) along with the corresponding attention weights produced by the softmax layer of the attention block. By inspecting individual filters and their associated attention weights, we could infer which features each filter emphasized. Representative examples of two traces are presented in Figure 2.

When analyzing individual filters, recurring patterns across different samples emerged. For example, in Figure 2a, filters 0 and 21 appear to capture the informative peaks in the signal, with the attention masks focusing on these regions. Filters 12 and 61 seem to concentrate on the noise after the translocation, while filter 1 incorporates both the baseline and noise information. Similarly, in Figure 2b, different filters highlight distinct aspects of the signal, including noise, peaks, and baseline parts.

To gain a broader understanding of the overall behavior of the attention mechanism, we computed the average of the filter outputs across all 64 filters and compared the result with the original input trace. This comparison, illustrated in Figure 3, provides an aggregated view of which signal components are emphasized by the model.

As shown in Figure 3, the averaged output contains peaks correlated to the barcode-related peaks in the original signal. Additionally, it appears to emphasize with peaks the end of the translocation event. Interestingly, for ambiguous samples (second row) where the barcode is not easily discernible from the raw trace, the averaged signal still delineates peaks that look more similar to the expected barcode. However, it is important to note that these are empirical observations and the internal representations learned by the network remain inherently opaque.

1.4 Tests to show accuracy increase

In this section, we present two different tests to verify that the proposed Brownian motion augmentation method increased the mean test accuracy. The results for the non-augmented runs correspond to the settings in the 8th row of Table 1, and the results for the augmented runs correspond to the settings in the 13th row of the same table. We define N as the number of runs used.

1.4.1 Probability of higher mean calculation

The first test consists of calculating the probability that the mean test accuracy is higher when trained with Brownian motion data augmentation, given the training results.

We define the random variable T as the test accuracy for a run without Brownian motion augmentation and T_a as the test accuracy when training with the augmentation method. Additionally, we introduce the random variable for their difference: $D = T_\text{a} - T$, and denote D_i for the ith training of the networks. Our mean estimator of the difference is defined by $\hat{\mu}_D = \frac{1}{N}\sum_{i=1}^{N} D_i$.

We rewrite the probability that the mean test accuracy of the augmented method is higher than that of the non-augmented method as

$$P(\mu_{T_\text{a}} > \mu_T | D_1, D_2 \ldots D_N) = P(\mu_{T_\text{a}} - \mu_T > 0 | D_1, D_2 \ldots D_N) = P(\mu_D > 0 | D_1, D_2 \cdots D_N).$$

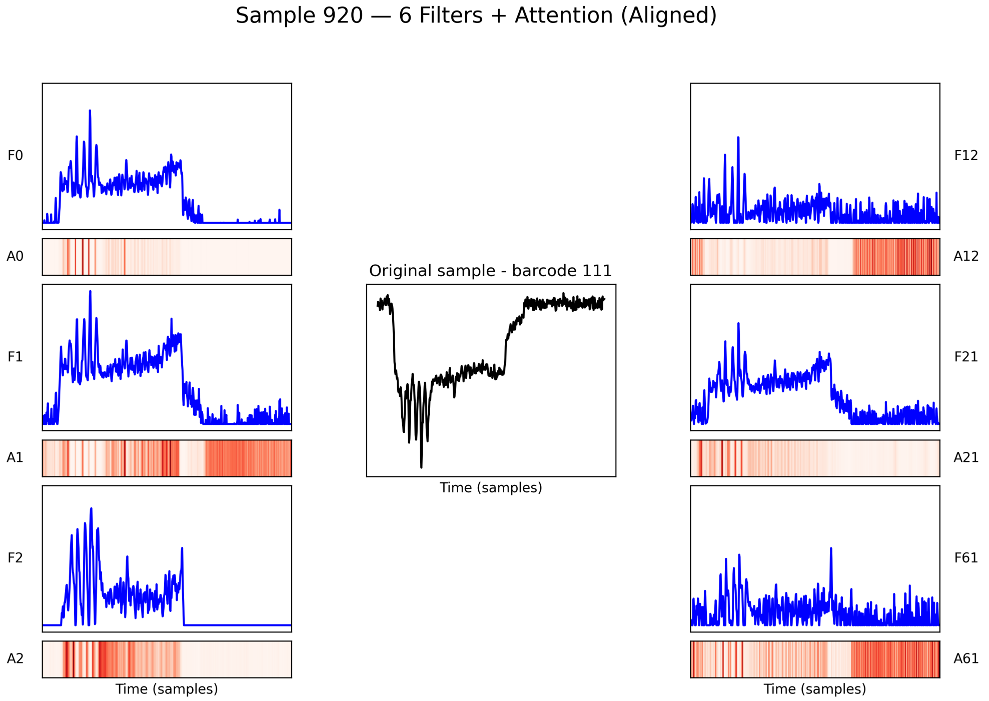

(a) Example of a trace with a clearly distinguishable barcode pattern.

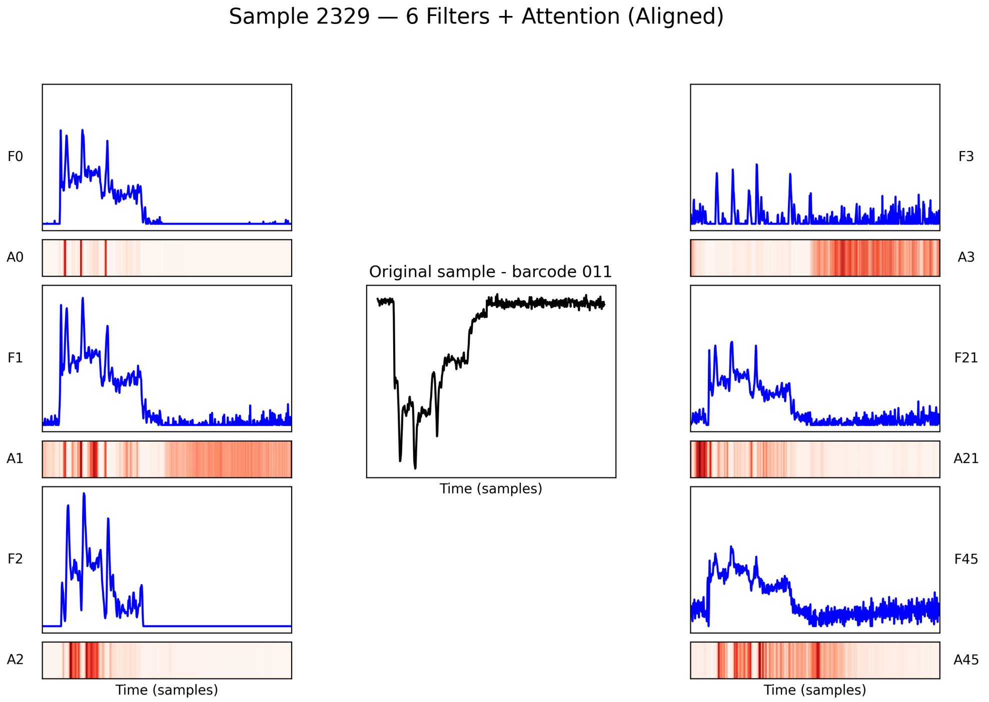

(b) Example of a trace with a barcode pattern that is difficult to discern.

Figure 2: Visualization of six selected filters and their corresponding soft attention masks for two input traces, as processed by the best YupanaNet model. Fn denotes the output of the nth filter, and An the associated soft attention weight.

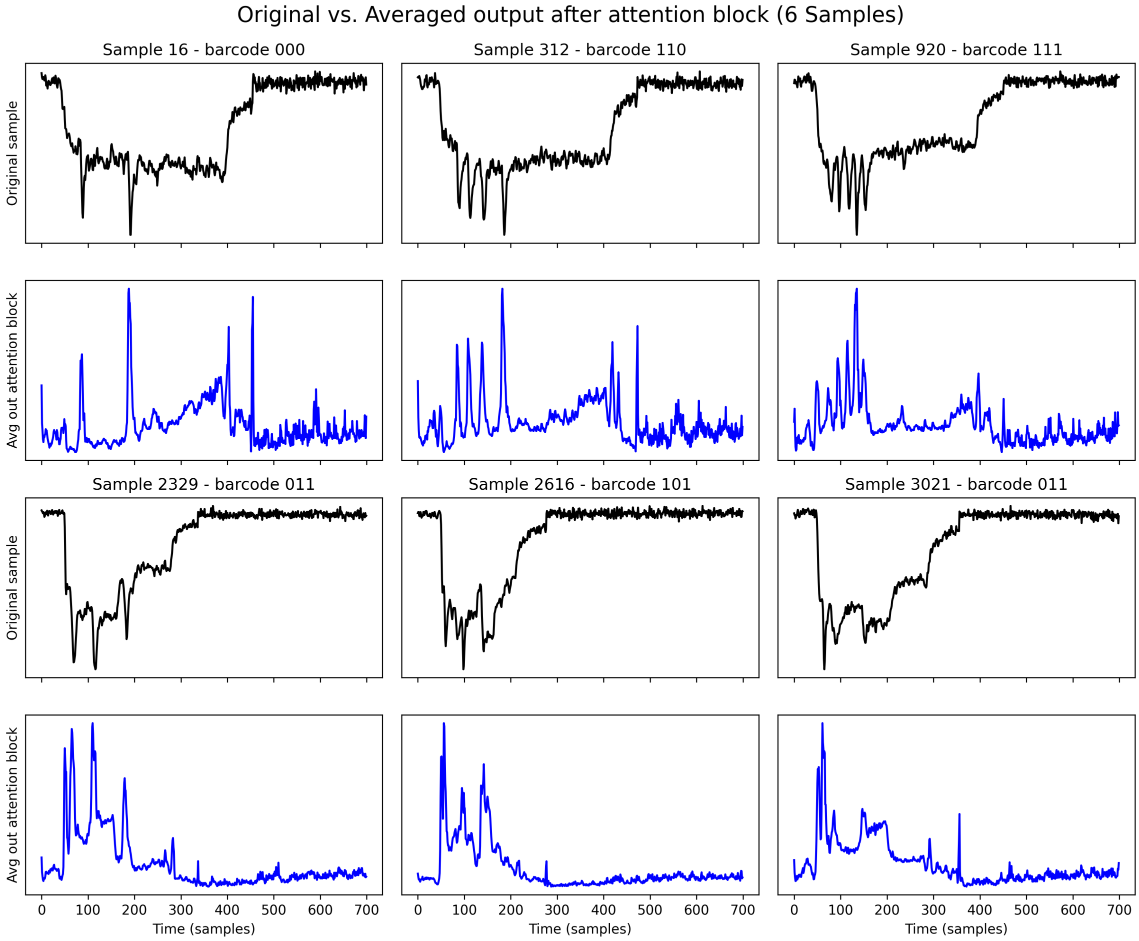

Figure 3: Original sample vs average output across filters after soft attention block.

Using the estimator $\hat{\mu}_D$ we can approximate the previous expression as $P(\hat{\mu}_D > 0)$. The distribution of $\hat{\mu}_D$ can be approximated to a normal distribution due to the central limit theorem. Since $\hat{\sigma}^2_{\hat{\mu}_D} = \frac{\hat{\sigma}^2_D}{N}$, we obtain that

$$P(\mu_{T_a} > \mu_T | D_1, D_2 \ldots D_N) = P(\hat{\mu}_D > 0) \simeq 1 - \Phi\left(-\frac{\hat{\mu}_D}{\hat{\sigma}_{\hat{\mu}_D}}\right)$$

where Φ represents the cumulative function of a standard normal distribution. After computing this expression with the results, we obtain a probability of 98.75% that the mean test accuracy increases when using our proposed data augmentation method.

1.4.2 P-value test

The second test involves rejecting the hypothesis that there is no improvement when training with Brownian motion data augmentation. We define our null thesis (H_0) as the Brownian motion augmentation does not improve the mean test accuracy. Subsequently, we derive the mathematical expression to be able to calculate the probability of the observations under the null hypothesis, and the low resulting probability allows us to reject the null hypothesis.

We introduce the binary random variable T_i, which represents whether the test accuracy was higher with Brownian augmentation than without it on the ith run. T_i takes the value 1 when the augmentation method increased the accuracy and 0 if it did not. Therefore, we model T_i as a uniformly distributed binary random variable ($\mu_{T_i} = 0.5$, $\sigma_{T_i} = 0.5$) when assuming that the null hypothesis is true. We introduce the random variable S as the sum of multiple T_i: $S = \sum_{i=1}^{N} T_i$. S is distributed as a binomial distribution with $p = 0.5$, $n = N$. Finally, we let s_e denote the empirically obtained sum of tests. The p-value, which is the probability of obtaining a result at least as extreme as the observed one under the null hypothesis, is given by

$$P(S \geq s_e | H_0) = \sum_{k=s_e}^{N} \binom{N}{k} 0.5^N.$$

Using the test accuracies from the experiments, we compute this probability, resulting in a p-value of 2%. This low p-value indicates that the observed improvement in test accuracy with Brownian motion augmentation is statistically significant, allowing us to reject the null hypothesis with considerable confidence.

1.5 Stretch-equivalent parameter estimation

This subsection details the calculation of the equivalent uniform stretch parameter of the Brownian motion data augmentation (σ_e). This augmentation method alters the event lengths, and our objective is to determine the relative standard deviation of the length variation. This parameter helps verify that uniformly stretching the data using σ_e does not increase accuracy, but using the Brownian motion data augmentation does.

In the methods section, we introduced the trace array X. Here, we define the function $l(X) : \mathbb{R}^L \to \mathbb{N}$, which estimates the length of the event. This function employs a straightforward approach using two threshold levels (h_s and h_e). The start of the event, i_s, is identified as the first instance when the trace falls below the start threshold h_s. The end of the event, i_e, is the last index where the trace value is below the end threshold h_e. Mathematically this can be expressed as

$$i_s(X) = min\ \{i : X[i] < h_s\},$$

$$i_e(X) = max\ \{i : X[i] < h_e\}.$$

And the event length is defined as

$$l(X) = i_e(X) - i_s(X).$$

While various length estimators could be proposed, our code demonstrates that this estimation is accurate for numerous samples. The thresholds used were $h_e = -0.25$ and $h_e = -0.5$

We denote the ith trace in our database as X_i and its Brownian motion augmented version as Y_i, with N_t being the total number of traces. To obtain the stretch equivalent parameter σe, first we calculate the mean stretch factor μ_e

$$\mu_e = \frac{1}{N_t} \sum_{i=1}^{N_t} \frac{l(X_i)}{l(Y_i)}.$$

Then, the variance σ_e^2 is

$$\sigma_e^2 = \frac{1}{N_t - 1} \sum_{i=1}^{N_t} \left(\frac{l(X_i)}{l(Y_i)} - \mu_e\right)^2.$$

After using these formulas in our dataset augmented with Brownian motion of $\sigma = 0.9$, we obtained that the equivalent stretch was $\sigma_e \simeq 0.05$.

1.6 YupanaNet tuning

As described in the methods section, the tuning of YupanaNet parameters was conducted without using any samples from the test dataset originally employed for QuipuNet. The original "train" dataset was split into training, validation, and test sets with 80%, 10% and 10% of the samples randomly selected, respectively. When discussing parameter tuning, the term "test set" refers to the samples from the original training dataset that were not used in the tuning trainings.

During each run for each neural network setting, the loss curves for the training and validation datasets were saved. Additionally, the accuracy results for the training, validation, and test datasets were recorded. In our analysis, we used the mean and standard deviation of all these results to compare different networks and select the optimal architecture for YupanaNet. The Adam optimizer [1] was utilized to fit the model.

Our initial model for tuning was QuipuNet with skip connections since QuipuNet was a state-of-the-art neural network, and extensive literature suggests that residual connections have potential accuracy improvements. We re-evaluated the parameters for augmentation steps, resulting in minor adjustments to the starting parameters for uniform stretching, noise addition, and magnitude multiplication.

Following the steps recommended by Yu and Zhu [2], we began by tuning the learning rate and subsequently selecting the batch size based on previous experiments. Next, we modified the architecture parameters. We first tuned the number of blocks and kernel sizes, testing several configurations with more blocks than QuipuNet. Then, we adjusted the number of units in the final dense layers. We also experimented with changing the activation functions from ReLUs to swish, but this did not result in any improvement. Following this, we compared different augmentation parameters, including the only parameter of the Brownian motion data augmentation, to fine-tune their performance. Finally, we introduced the attention layer to further enhance the network.

The different values tested during hyperparameter tuning can be found in the Jupyter notebooks of the repository of the paper. The final hyperparameters selected after tuning are summarized in Table 2, excluding the architectural parameters, which are detailed in the figures that illustrate the YupanaNet architecture.

Type	Parameter	Final value
Augmentation	Brownian motion driving std (σ)	0.8
Augmentation	Magnitude relative std	0.05
Augmentation	Stretching relative std	0.06
Augmentation	Noise std	0.02
Training	Learning rate	$2\,10^{-4}$
Training	Optimizer	Adam
Training	Number of epochs	100
Training	Early stopping patience	100

Table 2: Hyperparameter values after tuning

References

[1] Diederik P Kingma and Jimmy Ba. Adam: A method for stochastic optimization. *arXiv preprint arXiv:1412.6980*, 2014.

[2] Tong Yu and Hong Zhu. Hyper-parameter optimization: A review of algorithms and applications. *arXiv preprint arXiv:2003.05689*, 2020.
